# Molecular epidemiology and comparative genomics of carbapenemase-producing *Escherichia coli* isolates from 19 tertiary hospitals in China from 2019 to 2020

**DOI:** 10.3389/fmicb.2023.1056399

**Published:** 2023-04-21

**Authors:** Weihsin Ko, Songlu Tseng, Chiahsin Chou, Tianmeng Li, Rose Li, Yaqiao Zhang, Yun Li, Yuan Lv

**Affiliations:** ^1^Institute of Clinical Pharmacology, Peking University First Hospital, Beijing, China; ^2^Department of Trauma and Orthopaedics, Peking University People's Hospital, Beijing, China; ^3^Department of General Surgery, Peking University First Hospital, Beijing, China; ^4^Department of Infectious Diseases and Clinical Microbiology, Beijing Institute of Respiratory Medicine and Beijing Chao-Yang Hospital, Capital Medical University, Beijing, China; ^5^Department of Clinical Medicine, Civil Aviation General Hospital, Beijing, China; ^6^Department of Clinical Medicine, Peking University International Hospital, Beijing, China

**Keywords:** *Escherichia coli*, carbapenemase, molecular epidemiology, genetic characteristics, *bla*_*NDM*_ gene

## Abstract

**Background:**

The clinical use of carbapenems is facing challenges due to increased carbapenemase-producing *Escherichia coli* (CP-EC) infections over the past decade. Meanwhile, whole-genome sequencing (WGS) is an important method for bacterial epidemiological research. We aim to provide more gene-based surveys to explore the genomics and occurrence of CP-EC in China.

**Methods:**

A total of 780 *Escherichia coli* isolates were collected by the China Antimicrobial Resistance Surveillance Trial (CARST) from 2019 to 2020. An antibacterial susceptibility test was performed by using the agar dilution method. CP-EC were detected by the modified carbapenem inactivation method (mCIM), EDTA-modified carbapenem inactivation method (eCIM), and polymerase chain reaction (PCR). Homology analysis was performed by multilocus sequence typing (MLST). A conjugation experiment was performed to verify the transferability of plasmids carrying carbapenemase genes. WGS was conducted to explore the gene-environment of the carbapenemase gene.

**Result:**

Of the 780 *Escherichia coli* isolates, 31 isolates were insensitive to carbapenem with a rate of 4%. Among them, 13 CP-EC isolates had transferability of the *bla*_*NDM*_ gene. These isolates belonged to nine distinct sequence types (STs), with some correlation. We found that two (2/13, 15.4%) of the CP-EC isolates that were collected from blood specimens were highly pathogenic and also showed high transferability of the *bla*_*NDM*_ gene. In addition, eight (8/13, 61.5%) of the CP-EC isolates were found to be multidrug-resistant.

**Conclusion:**

With the increasing use of carbapenem, CP-EC isolates accounted for nearly half of the total carbapenem-insensitive *Escherichia coli* isolates. Our findings highlight the urgent need to pay attention to CP-EC isolates in bloodstream infections and ESBL-producing CP-EC isolates. Based on the One Health concept, we suggest various measures, including the development of bacterial vaccines, antibiotic management, and establishment of better medical environments, to avoid the outbreak of CP-EC.

## 1. Introduction

Carbapenems are considered one of the last resort antibiotics against extended-spectrum beta-lactamase-producing gram-negative bacterial infections (Li et al., 2021). However, carbapenem resistance has become a major public health issue, with the World Health Organization listing carbapenem-resistant *Enterobacteriaceae* as the priority bacteria. *Escherichia coli* (*E. coli*), which can cause urinary, respiratory, and bloodstream infections in addition to other pathogens in immunocompromised individuals (Tao et al., 2020), is primarily responsible for community-associated infections, making it difficult to implement traditional preventive measures based on hospital-acquired infections. In China, the isolation rates of carbapenem-resistant *Escherichia coli* (CRECO) have shown an increasing trend in recent years, despite being lower than in previous years. The China Antimicrobial Resistance Surveillance trail (CARST) reported that between 2016 and 2020, the average isolation rates of CRECO were 1.5, 1.5, 1.5, 1.7, and 1.6%, respectively. Although the 2020 isolation rate of CRECO decreased by 0.1% points compared to 2019, it is still higher than the average rate in previous years. Furthermore, the isolation rates of CRECO also varied among regions, with Henan Province showing the highest isolation rates in 2016 and 2018 (3 and 2.9%), and Qinghai Province showing the lowest (0.6 and 0.2%). In 2017, Liaoning Province showed the highest isolation rates (2.8%), while the Tibet Autonomous Region showed the lowest (0.3%). In 2020, Beijing had the highest isolate rates of CRECO (3.2%), while the Tibet Autonomous Region had the lowest proportion (0.2%).

Carbapenem resistance is a growing public health concern due to its widespread dissemination facilitated by carbapenemase, a crucial factor responsible for its transmission. The transfer of genes encoding carbapenemase through conjugative plasmids, transposons, and insertion sequences has led to horizontal transmission (Conlan et al., 2014). In China, carbapenemase-producing *Enterobacterales* (CPE) infections have increased over the past decade, with *E.coli* being the second most common CPE (Deng et al., 2022; Wang et al., 2022). Notably, a majority (76.2%, 16/21) of carbapenemase-producing *Escherichia coli* (CP-EC) cases were detected in a hospital in Shanghai (Zhang et al., 2015). In addition, 35.8% of extended-spectrum β-lactamase-producing *Escherichia coli* (ESBL-EC) and 0.9% of CP-EC were isolated from the feces of healthy children's feces in south-central China communities (Liu et al., 2022). Tracing the genetic environment and epidemiology of carbapenemase genes is paramount to public health and aids in designing infection management and prevention strategies.

Polymerase chain reaction (PCR) and pulsed-field gel electrophoresis (PFGE) are traditional techniques used in molecular epidemiological research of antimicrobial resistance. However, these techniques have limitations, and whole-genome sequencing (WGS) can be a more effective tool for analyzing antimicrobial resistance. WGS can directly assemble the sequence fragments without designing specific primers to obtain the whole genome of the isolate. The isolate's species, antibiotic resistance genes, virulence factors, and mobile genetic elements can be determined by comparing the whole genome of the isolate with the database. Moreover, genome comparisons of multiple isolates can provide information on the molecular epidemiology of specific resistance genes or mechanisms of antimicrobial resistance. Therefore, WGS and bioinformatics analysis can be more effective in assisting with molecular epidemiological research of antimicrobial resistance. Despite the gene-environment of plasmids has been studied recently, nationwide WGS-based epidemiology of clinical CP-EC has not yet been carried out in China. As a result, we aim to provide more gene-based investigations to explore the genomics and occurrence of CP-EC in China.

## 2. Materials and methods

### 2.1. Bacterial strain identification and antimicrobial susceptibility testing

A total of 780 *E. coli* isolates were collected between July 2019 and June 2020 from 19 tertiary hospitals by the China Antimicrobial Resistance Surveillance Trial (CARST) and were preserved by the Institute of Clinical Pharmacology of Peking University. Samples processing, microbial identification, and antibiotic susceptibility testing were performed by the microbiology laboratories of the 19 hospitals, according to a shared protocol. According to CLSI-M100-S31, antibiotic susceptibility testing (AST) was carried out by the reference broth microdilution method. Antimicrobial agents or combinations tested included cefotaxime, ceftazidime, cefepime, aztreonam, imipenem, meropenem, ertapenem, amikacin, minocycline, tigecycline, ciprofloxacin, and polymyxin E. *E. coli* ATCC25922 was used as the control strain for AST. All tests were performed in duplicate, and three biological replicates per strain were included in each test. PCR was used to amplify the 16SrRNA of the isolates, and the amplified products were sent to Beijing SinoGenoMax Co., Ltd. for sequencing. The isolates were confirmed to be *E. coli* by comparing the sequences in NCBI (https://blast.ncbi.nlm.nih.gov/).

### 2.2. Detection of carbapenemase phenotypes and genes, and transferability of carbapenemase gene

To detect carbapenemase phenotypes, we employed the modified carbapenem inactivation method (mCIM) and EDTA-modified carbapenem inactivation method (eCIM), and *Klebsiella pneumoniae* ATCC BAA-1705 and *Klebsiella pneumoniae* ATCC BAA-1706 were utilized as quality controls. We targeted the following carbapenemase genes for PCR detection: *bla*_*KPC*_*, bla*_*SME*_*, bla*_*IMI*_*, bla*_*GES*_*, bla*_*IMP*_*, bla*_*VIM*_*, bla*_*SIM*_*, bla*_*GIM*_*, bla*_*NDM*_, and *bla*_*OXA*−48_. To assess the transferability of *bla*_*NDM*_, a conjugation assay was performed on the 13 *bla*_*NDM*_-positive *E. coli* isolates, using sodium azide-resistant *E. coli* J53 as the recipient strain. *E. coli* isolates were analyzed based on genomic sequences using *in silico* MLST and cgMLST analysis on the EnteroBase online platform (http://enterobase.warwick.ac.uk) for *E. coli*. Seven housekeeping gene loci, including *adk, fumC, gyrB, icd, mdh, purA*, and *recA*, were chosen for MLST analysis of *E. coli*. The cluster analysis is performed by Bionumerics software v.7.0 based on MLST information.

### 2.3. Whole-genome sequencing and analysis

We performed whole-genome sequencing (WGS) of CP-EC isolates using the Nanopore *De Novo* platform and Circular Library. We prepared the sequencing library using the rapid sequencing kit (Oxford Nanopore Technologies) and subjected the sequencing data to several processing steps. First, we checked the consistency of each base in the original data and assigned a quality value to each base based on the results of the consistency check. Next, we removed residual adapters using strict parameters and filtered out regions with Q20 < 0.8 in the reads to obtain high-quality subreads sequences. These high-quality subreads sequences were then used for subsequent analysis. We assembled the high-quality subreads using the hierarchical genome assembly process (HGAP) 3.0 software, which corrects errors in long reads with short reads. The resulting assembly was further processed using the classic OLC algorithm architecture to obtain the final assembly. To identify transposon sequences, we compared the assembly results with known transposon sequence libraries using RepeatMasker software (using the Repbase database) and RepeatProteinMasker software (using the transposon protein library that comes with RepeatMasker). We also used tandem repeat finder (TRF) software to predict tandem repeats. Contigs were screened for plasmid and resistance gene content using PlasmidFinder and ResFinder tools at the Center for Genomic Epidemiology (CGE) server (http://www.genomicepidemiology.org/services/), respectively. To identify virulence genes related to CP-EC isolates, we utilized VirulenceFinder at CGE and the VFDB database. In addition, we used ISFinder to detect insertion sequences with a threshold of e value1e-5 and generated a gene alignment diagram using Easyfig.

## 3. Results

### 3.1. Incidence of carbapenemase gene in carbapenem-resistant *Escherichia coli*

**Figure 1:**
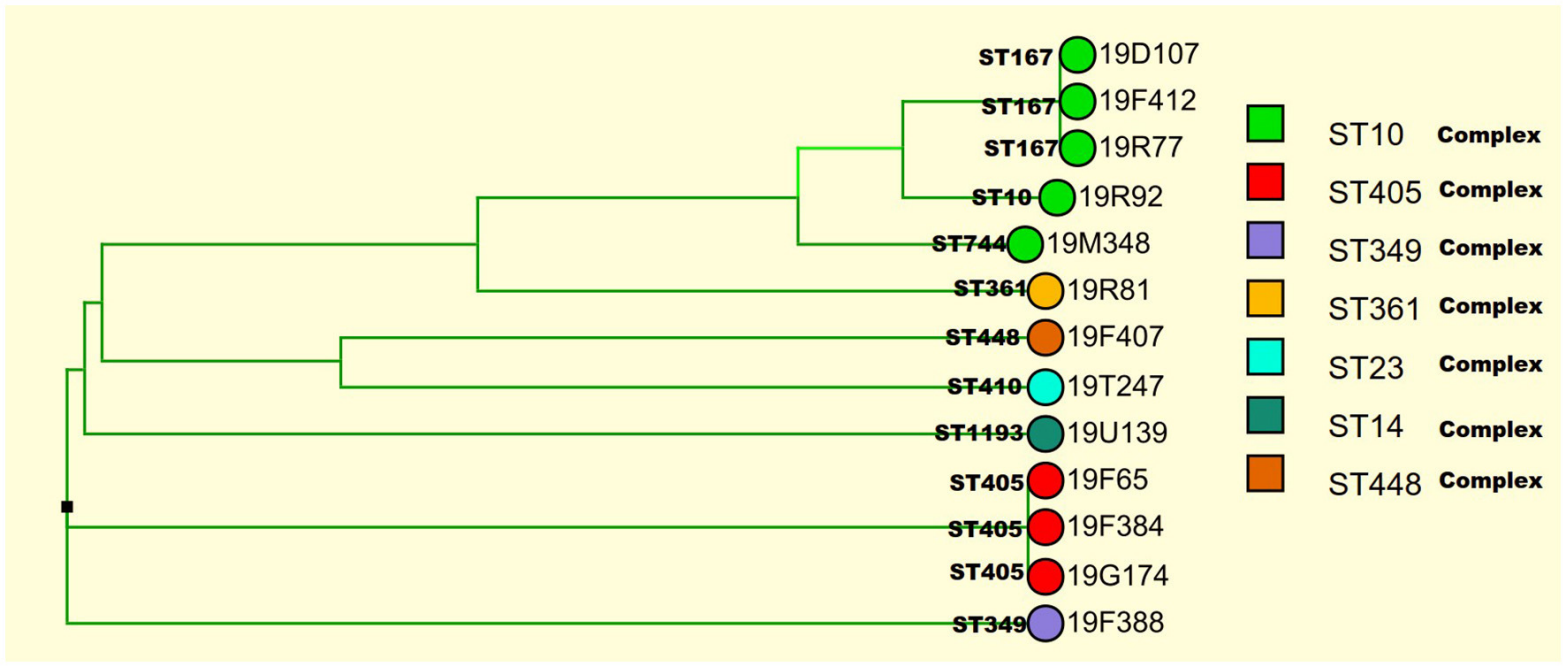
Identification of 13 CP-EC lineages using genome-based phylogenetic analysis.

A total of 31 *E.coli* isolates displayed insensitive to meropenem, imipenem, or ertapenem. Among 31 insensitive *E. coli* isolates, the mean minimal inhibitory concentrations (MICs) of meropenem against 50% (MIC_50_) and 90% (MIC_90_) were 0.25 and 32 μg/ml, respectively. The MIC_50_ and MIC_90_ values of imipenem were 0.5 and 16 μg/ml, respectively. The MIC_50_ and MIC_90_ values of ertapenem were 4 μg/ml and 128 μg/ml, respectively. Of the 31 isolates, 13 (41.9%), 14 (45.2%), and 30 (96.7%) were insensitive to meropenem, imipenem, and ertapenem, respectively. In addition, 96% (29/31) of isolates exhibited resistance to third/fourth-generation cephalosporins. Clinical information about the 31 *E. coli* isolates is presented in Table 1. Thirteen carbapenemase-producing *E. coli* (CP-EC) isolates were confirmed to produce metallo-β-lactamase using the modified carbapenem inactivation method (mCIM) and EDTA-modified carbapenem inactivation method (eCIM). Of these 13 CP-EC isolates, only *bla*_*NDM*_ was detected, without any other carbapenemase genes. Among these, nine isolates (69.2%) carried *bla*_*NDM*−5_, three isolates (23.1%) carried *bla*_*NDM*−1_, and one isolate (7.7%) carried *bla*_*NDM*−7_. The genome-based phylogenetic analysis revealed the presence of nine CP-EC lineages: ST167 (3 isolates), ST405 (3 isolates), ST744, ST10, ST361, ST1193, ST488, ST410, and ST349 (refer to Figure 1 and Table 2 for details).

**Table 1:** Clinical information of 31 *Escherichia coli* isolates.

**Isolates**	**Gender**	**Age**	**Age classification**	**Year**	**Specimen classification**
19D107	Male	17 days	Children	2019	Secretion
19F065^*^	Male	60	Adult	2019	Sputum
19R077	Male	79	Elder	2019	Cerebrospinal fluid
19R081	Female	51	Adult	2019	Bloodstream
19R092	Male	36	Adult	2019	Secretion
19U139	Male	53	Adult	2019	Bloodstream
19F053	Male	61	Adult	2019	Drainage
19F054	Male	52	Adult	2019	Bloodstream
19G152	Male	74	Elder	2019	Bloodstream
19M056	Male	35	Adult	2019	Urine
19O014	Female	78	Elder	2019	Bloodstream
19O017	Male	27	Adult	2019	Drainage
19P164	Female	40	Adult	2019	Bloodstream
19U138	Female	77	Elder	2019	Bile
19W025	Female	51	Adult	2019	Secretion
19F384	Female	65	Elder	2020	Bloodstream
19F388	Male	82	Elder	2020	Bile
19F407	Male	12	Children	2020	Bloodstream
19F412	Male	33	Adult	2020	Bloodstream
19G174	Female	75	Elder	2020	Bloodstream
19M348	Male	66	Elder	2020	Drainage
19T247	Male	38	Adult	2020	Bloodstream
19F395^*^	Female	84	Elder	2020	Bloodstream
19G160	Female	26	Adult	2020	Bloodstream
19G179	Female	66	Elder	2020	Bloodstream
19K198	Female	35	Adult	2020	Drainage
19O191	Female	88	Elder	2020	Bloodstream
19Q278	Female	51	Adult	2020	Urine
19Q287^*^	Male	54	Adult	2020	Drainage
19R296	Female	83	Elder	2020	Urine
19R360^*^	Male	75	Elder	2020	Secretion

The age groups in this study were defined as follows: children, defined as patients aged 14 years or younger, and elders, defined as patients aged 65 years or older. ICU sources are indicated by an asterisk (^*^) in the table.

**Table 2:** Phylogenetic analysis and plasmid content of 13 CP-EC isolates.

**Isolates**	**NDM type**	**Sequence type (ST)**	**Plasmid type**	**Plasmid length**
19F065	*bla_*NDM*−7_*	ST405	IncX3	46,159 bp
19F388	*bla_*NDM*−1_*	ST349	IncX3	56,904 bp
19F407	*bla_*NDM*−5_*	ST448	IncX3	46,158 bp
19M348	*bla_*NDM*−1_*	ST744	IncX3	54,035 bp
19R077	*bla_*NDM*−5_*	ST167	IncX3	46,163 bp
19R081	*bla_*NDM*−5_*	ST361	IncX3	48,787 bp
19R092	*bla_*NDM*−5_*	ST10	IncX3	45,071 bp
19T247	*bla_*NDM*−5_*	ST410	IncX3	52,884 bp
19U139	*bla_*NDM*−1_*	ST1193	IncN	54,734 bp
19F412	*bla_*NDM*−5_*	ST167	IncI	268,028 bp
19D107	*bla_*NDM*−5_*	ST167	IncFII	95,105 bp
19F384	*bla_*NDM*−5_*	ST405	IncFII	95,073 bp
19G174	*bla_*NDM*−5_*	ST405	IncFII	91,572 bp

The clonal relationship between CP-EC ST448 and ST410 was found to be highly related, with ST410 originating from ST448. In addition, the three NDM-1 producing CP-EC isolates were classified under ST349, ST744, and ST1193, respectively. Notably, NDM-7 producing CP-EC isolate, 19F065, was assigned to ST405. Furthermore, ST167, ST10, and ST744 exhibited close relatedness, with ST167 and ST744 emerging as derivatives of ST10. These findings suggest a complex evolutionary history of CP-EC.

### 3.2. Plasmid content of carbapenemase-producing *E. coli*

The successful transfer of the carbapenemase gene to *E. coli* J53 recipients was observed in all 13 isolates, with a minimum of a 32-fold increase in the MIC of carbapenems for the transconjugants. Plasmid content was evaluated by PlasmidFinder and is presented in Table 2. IncX3 was the predominant plasmid type observed in CP-EC (8/13, 61.5%), followed by IncFII (3/13, 23.1%). The average length of the plasmids was 50,000 bp, of which p19D107 and p19F412 were longer, with a length of 95,105 bp and 268,028 bp, respectively.

### 3.3. Genomic analysis of carbapenemase-producing *E. coli*

The plasmids of the 13 CP-EC isolates were found to harbor mobile genetic elements and insertion sequences (ISs), as determined by genomic analysis. IS*Aba125* was frequently located upstream of the genomic region (5/13, 38.5%), while IS*15* was frequently located downstream (9/13, 69.2%). The plasmid carrying *bla*_*NDM*−7_, specifically p19F065, had a similar genetic environment to those carrying *bla*__*NDM*−5__, such as p19F407 and p19R077. Notably, among the nine isolates carrying *bla*_*NDM*−5_, p19R077 and p19F407 exhibited nearly identical genetic environments, with IS*Aba125*, IS*C1041*, and IS*5B* detected upstream of *bla*_*NDM*−5_.

#### 3.3.1. Analysis of carbapenemase gene-environment

**Figure 2:**
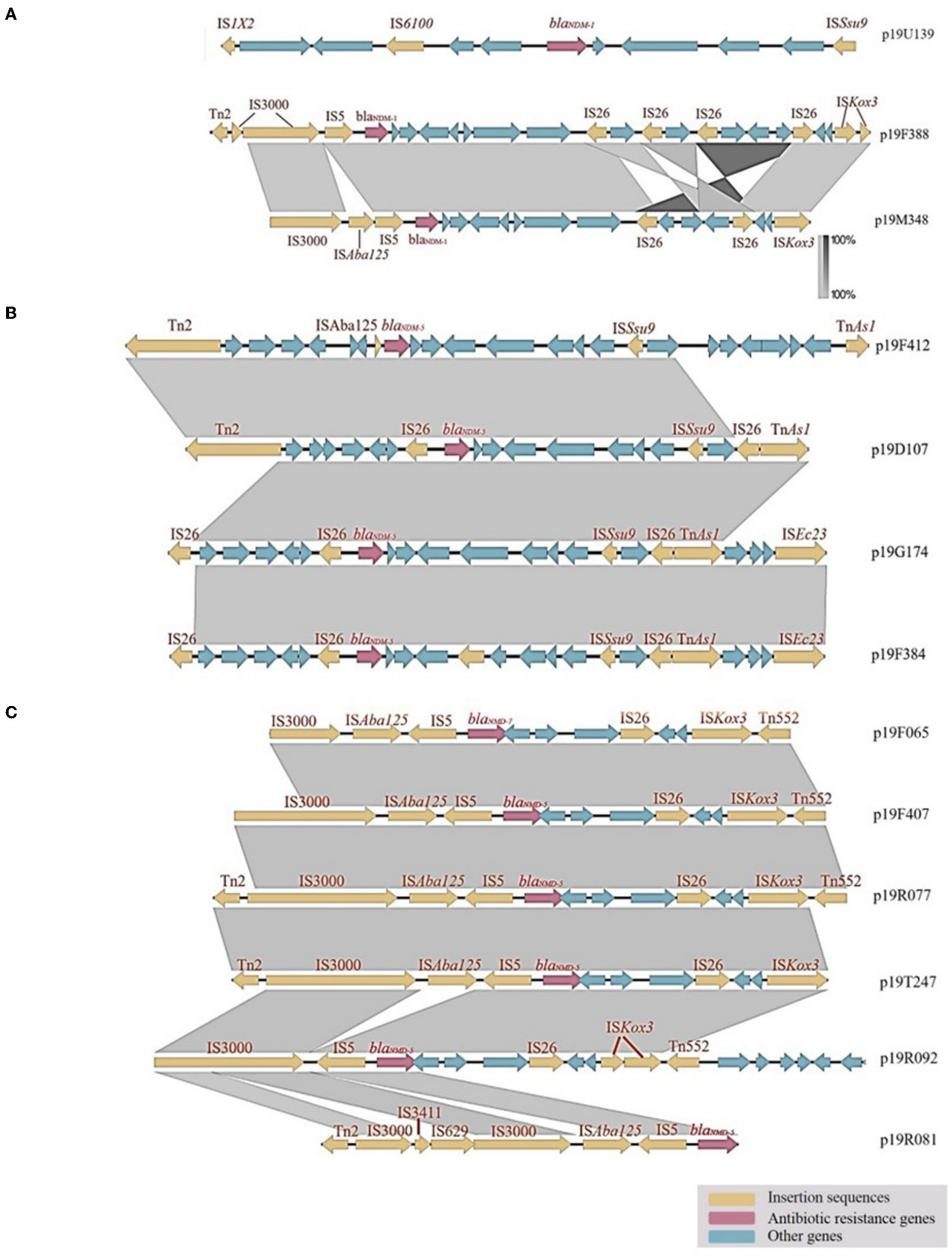
Genetic analysis of carbapenemase-producing *E. coli* isolates: Insights from gene alignment and gene-environment analysis of *bla*_*NDM*−1_, *bla*_*NDM*−5_, and *bla*_*NDM*−7_ in different plasmids. **(A)** The gene-environment of p19U139 was found to differ from that of other plasmids, with IS*6100* and IS*1X2* belonging to the IS*6* and IS*1* transposase families, respectively. **(B)** In the IncFII plasmid, IS*26* was found to carry both *bla*_*NDM*−5_ and *emrE*, a gene related to an efflux protein. **(C)** Analysis of *bla*_*NDM*−5_ and *bla*_*NDM*−7_ gene-environment in IncX3 plasmids.

The plasmid p19U139, classified as an IncN plasmid, carries the carbapenemase gene *bla*_*NDM*−1_. IS*1X2* and IS*61009* were identified upstream of p19U139, while IS*Ssu9* was found downstream. Meanwhile, the plasmids p19F388 and p19M348, which also carry *bla*_*NDM*−1_, were classified as IncX3 plasmids. Specifically, p19F388 contained two IS*3000* elements, one *Tn2* transposon, and one IS*5* element, while p19M348 had an IS*Aba125* insertion between IS*3000* and IS*5*. These two plasmids shared a similar downstream gene-environment, consisting of an IS*Kox3* element belonging to the IS*L3* transposase family and multiple IS*26* elements of the IS*6* transposase family, which carried the *bla*_*SHV*−2_ gene at a distance. According to the gene alignment diagram, the core structure of the IncX3 plasmid is diverse by carrying different *bla*_*NDM*_ subtype genes. For instance, the core structures of IncX3 plasmid carrying the *bla*_*NDM*−1_ and *bla*_*NDM*−5_ genes are IS*3000*-IS*5*-*bla*_*NDM*−1_-IS*26*-IS*Kox3* and IS*3000*-IS*Aba125*-IS*5*-*bla*_*NDM*−5_-IS*26-*IS*Kox3*, respectively. On the other hand, the IncFII plasmid carrying the *bla*_*NDM*−5_ gene has a different core structure: IS*26*-*bla*_*NDM*−5_-IS*Ssu9*-IS*26*-Tn*As1*. For further details, refer to Figure 2A.

#### 3.3.2. Analysis of the *bla*_**NDM*−5*_ gene-environment in the IncFII plasmid

The gene-environments of the IncFII plasmids, namely p19D107, p19G174, and p19F384, show a high degree of similarity, as all three contain an adjacent reverse IS*26* element located upstream of the *bla*_*NDM*−5_ gene. In addition, these plasmids possess IS*26*, ISSsu9, and TnAs1 elements at a distance from the *bla*_*NDM*−5_ gene (refer to Figure 2B). Conversely, p19F412 is a longer IncI1-I plasmid that shares a partially similar gene-environment with p19D107.

#### 3.3.3. Analysis of *bla*_**NDM*−5*_ and *bla*_**NDM*−7*_ gene-environment in IncX3 plasmids

p19F065, p19F407, p19R077, and p19T247 are all IncX3 plasmids with the same upstream and downstream gene-environments. These plasmids have a segment of IS3000, IS*Aba125*, and IS*5* upstream and a segment of IS*26*, IS*Kox3*, and Tn*552* downstream. p19R092 has a missing IS*Aba125* upstream, and IS*Kox3* downstream was divided into two segments. The *bla*_*NDM*−5_ gene in p19R081 is located at the edge of the genome, so the downstream environment is unknown. Unlike other plasmids, p19R081 has IS*3411* and IS*629*, dividing IS*3000* into two segments (Figure 2C).

#### 3.3.4. Resistome and virulence genes of carbapenemase-producing *E. coli*

**Figure 3:**
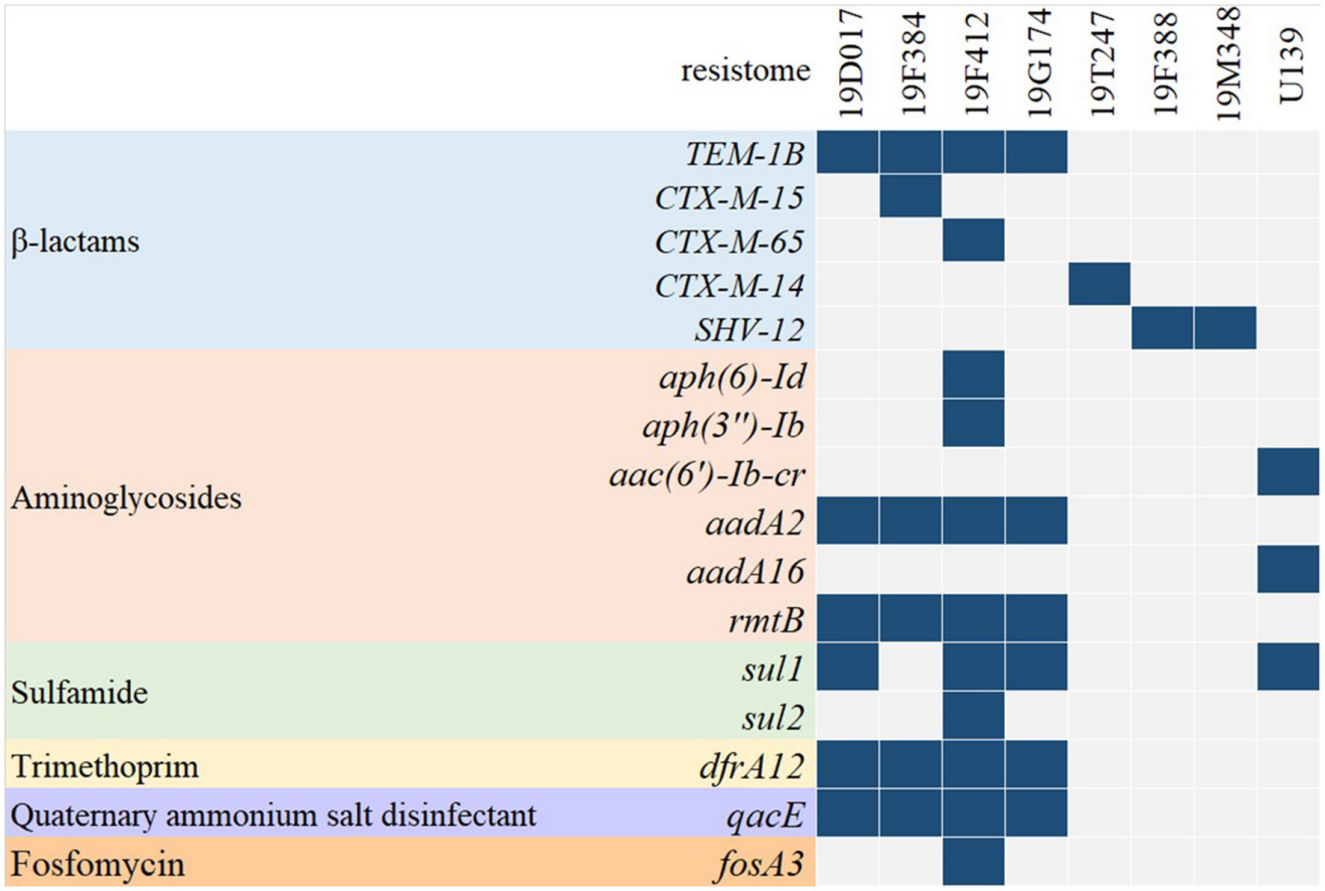
Comprehensive analysis of the resistome and prevalence of ESBL genes in CP-EC isolates.

The resistome of CP-EC isolates is visualized in Figure 3, revealing the presence of 16 antibiotic resistance genes linked to various antibiotic classes, including β-lactams, aminoglycosides, sulfamide, trimethoprim, quaternary ammonium salt disinfectant, and fosfomycin. Notably, five isolates (5/13, 38.5%) were found to carry extended-spectrum β-lactamase (ESBL) genes, among which *bla*_*SHV*−12_ and *bla*_*CTX*−*M*_ were identified in two and three isolates, respectively.

**Figure 4:**
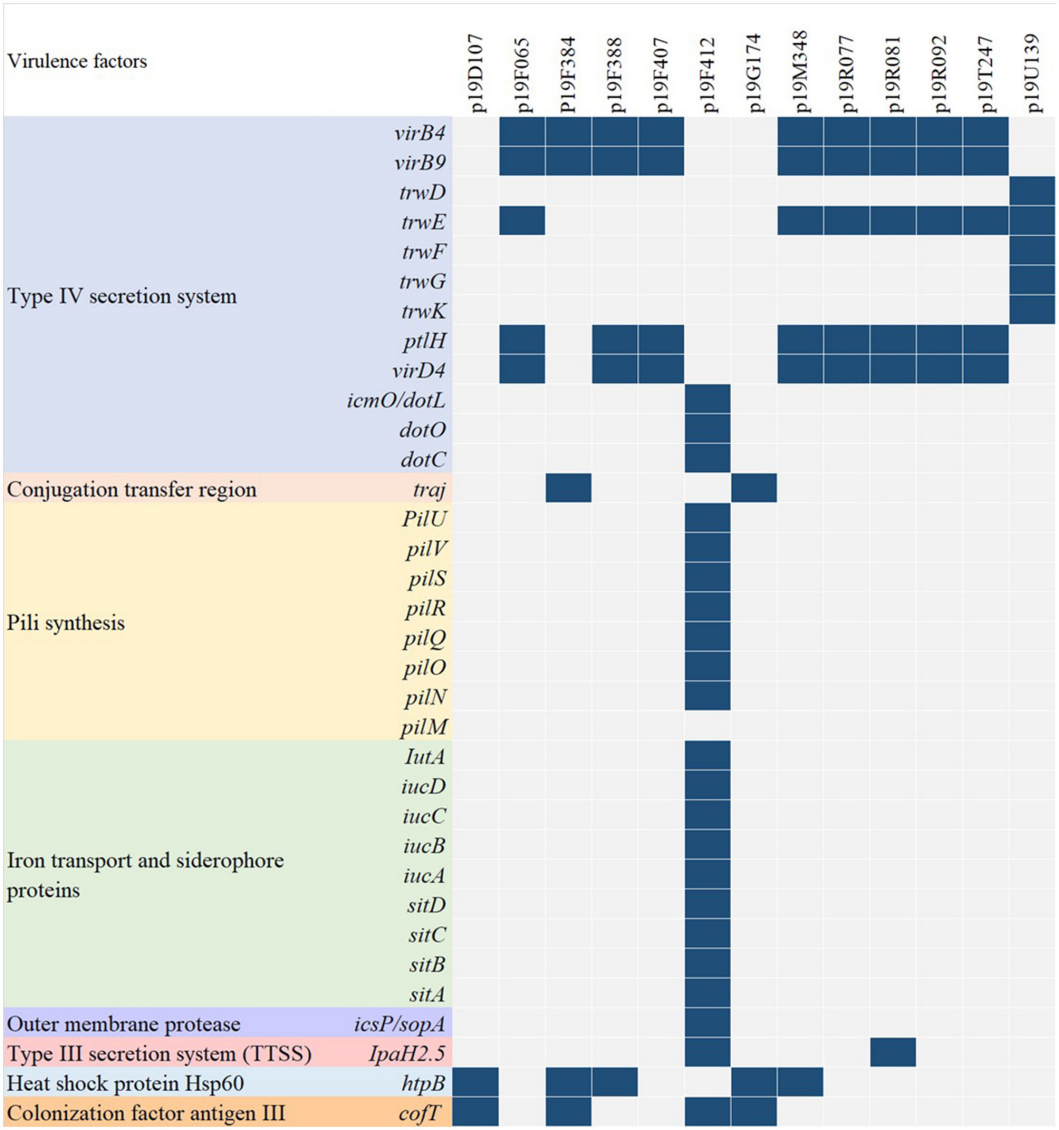
Variable distribution and characterization of virulence genes in CP-EC isolates.

Virulence genes were detected in each isolate with a variable distribution (Figure 4). The majority of isolates (10/13, 76.9%) harbored sequences encoding type IV secretion system (T4SS), specifically *virB4, virB9, trwD, trwE, trwF, trwG, trwK, ptlH, virD4, icmO/dotL, dotO*, and *dotC* (Christie et al., 2014). In addition, the *traj* gene, related to the plasmid conjugation transfer region, was found in one isolate (Liu G. et al., 2019). In the p19F412 isolate, several sequences encoding adhesion proteins were identified, including *PilU, pilV, pilS, pilR, pilQ, pilO, pilN*, and *pilM*, involved in pili synthesis (Foley et al., 2021); *IutA, iucD, iucC, iucB, iucA, sitD, sitC, sitB*, and *sitA*, encoding iron transport and siderophore proteins (da Cruz Campos et al., 2020); and *icsP/sopA*, associated with outer membrane protease (Tran et al., 2013). Furthermore, the type III secretion system (TTSS) invasion antigen, *IpaH2.5*, was identified in 19F412 and 19R081 (Tran et al., 2013), while the *htp* operons, a molecular chaperone related to heat shock protein Hsp60, were present in four isolates. The colonization factor antigen III-encoding *cofT* genes, facilitating bacterial adherence to the host, were identified in three isolates. Interestingly, IncX3 plasmids carried several genes associated with T4SS, while IncFII plasmids did not possess genes related to TTSS or T4SS. Remarkably, p19F412 and p19R081 isolates contained genes associated with both TTSS and T4SS.

## 4. Discussion

The prevalence and impact of CPE vary globally. In the United States, CPE is responsible for approximately 9,300 hospital-acquired infections annually, and approximately 50% of patients with bloodstream infections (BSIs) caused by CPE ultimately succumb to the infection (Villegas et al., 2016). Meanwhile, in central China, a surveillance report identified *E. coli* as one of the main pathogens causing bloodstream infections (Tian et al., 2018). In our study, we found that CP-EC from blood specimens accounted for 53.8% (7/13) of the total CP-EC, with *bla*_*NDM*−5_-producing isolates making up the majority, two of which belonged to ST405. These isolates were capable of transmitting carbapenemase genes through IncFII, IncI, IncN, and IncX3, with p19F412 and p19R081 carrying both TTSS and T4SS, making them highly transferable and pathogenic.

The dissemination of CPE isolates is a serious concern as these infections require aggressive and prolonged antibiotic therapy, leading to an increased risk of adverse events and prolonged hospitalization. A recent investigation in Australia found that patients colonized with CP-EC had a significantly longer hospital stay and higher medical costs during an outbreak (Rodriguez-Acevedo et al., 2020). These findings underscore the importance of managing and preventing future outbreaks caused by CP-EC.

The co-occurrence of carbapenemases with other β-lactamases, such as TEM, SHV, or CTX-M, confers resistance in CPE to almost all β-lactam antibiotics (De Angelis et al., 2020). In our study, 61.5% of the 13 isolates belonged to multidrug-resistant CP-EC, with seven isolates producing TEM, CTX-M, and SHV. The co-occurrence of these resistance mechanisms can pose challenges in clinical laboratories since carbapenemases are not inhibited by clavulanic acid, which is used in the standard double-disk test for identifying ESBLs recommended by the Clinical and Laboratory Standards Institute (CLSI). As a result, detecting multiple β-lactamases in CP-EC can be difficult, which may lead to heightened transmission and outbreaks in healthcare settings and community-acquired infections. The increased use of carbapenems has led to the gradual detection of CP-EC isolates in the stool samples of healthy individuals in the community (Bezabih et al., 2021). Furthermore, CP-EC isolates are not restricted to human communities or healthcare settings and are increasingly being detected in food-producing animals (Bonardi and Pitino, 2019). CTX-M-producing *E. coli* was the predominant multidrug-resistant *E. coli* isolates in food-producing animals (Michael et al., 2017), with ESBL-producing *E. coli* isolates from pigs in China mainly producing CTX-M (Tian et al., 2009). In addition, CTX-M-14, CTX-M-55, and CTX-M-65 were the most prevalent genotypes among CTX-M-producing *E. coli* isolates from food animals in China (Rao et al., 2014). These isolates may be transmitted to humans through food consumption or contact with animals and can cause serious infections that are difficult to treat. The overuse of antibiotics in food-producing animals has been identified as a major driver of antibiotic resistance, and the detection of CP-EC isolates in these animals further underscores the urgent need for better antibiotic stewardship practices.

We identified three CTX-M-producing isolates that carried multidrug resistance genes by IncI and IncX3. IncI plasmids have been found to facilitate the transmission of the *bla*_*CTX*−*M*_ between poultry and humans (Kopotsa et al., 2019), highlighting their potential as vectors for expanded carbapenemase gene transmission. Furthermore, IncI plasmids have been shown to promote transmission between mothers and newborns, suggesting that they may increase the transmission of carbapenemase genes in neonatal infections (Hagb et al., 2020).

The clonal relationship analysis revealed that the spread of 13 NDM-producing *E. coli* was polyclonal, with common *E. coli* sequence types such as ST167 and ST405 carrying most *bla*_*NDM*−5_. However, rare *E. coli* sequence types, such as ST744, ST349, and ST1193, were found to carry *bla*_*NDM*−1_. Among the plasmids observed in CP-EC, IncX3 was the predominant plasmid type, accounting for 61.5% (8/13) of isolates. The remarkable stability and efficient horizontal transfer of the IncX3 plasmid can be attributed to its low fitness cost, which can enhance its potential to spread between bacterial populations (Ma et al., 2020). In transconjugants, the presence of the IncX3 plasmid resulted in significantly higher levels of carbapenem resistance and *bla*_*NDM*−5_, compared to transconjugants carrying both plasmids or only the IncFII plasmid (Yang et al., 2020). Notably, studies have demonstrated that the IncX3 plasmid can mediate the transfer of *bla*_*NDM*−5_genes among different *Enterobacter* species over a wide range of temperatures (Liu Z. et al., 2019). The IncX3 plasmid is not only the most prevalent vehicle for *bla*_*NDM*−5_ but also a critical platform for the evolution of *bla*_*NDM*−5_, which has led to the emergence of novel NDM variants (Wu et al., 2019). Our findings indicate that the presence of IncX3 plasmid is widespread among CP-EC, which could further limit medication options and exacerbate the burden on the healthcare system.

Our study had several limitations that should be acknowledged. First, while we were able to access the specimens for the CP-EC, we did not have access to information on the patient's diagnosis or antibiotic prescriptions. This lack of information may have impacted our ability to fully understand the epidemiology and clinical significance of CPE infections. Second, although we included hospitals from various regions throughout China, the sample size was limited, which may have restricted our ability to generalize our findings to the entire population of China. Therefore, future studies should focus on collecting more comprehensive clinical data and engage in longer-term surveillance to enable a more in-depth investigation of CPE prevalence and characteristics across China. By doing so, we can gain a more comprehensive understanding of this emerging public health threat and develop effective strategies to control its spread.

## 5. Conclusion

With the increasing use of carbapenem, CP-EC clinical isolates accounted for nearly half of the total CRECO clinical isolates. We need to pay more awareness to CP-EC in BSI because the isolates were transferable and pathogenicity, which increases the burden on patients and medical settings. Currently, ESBL-producing CP-EC isolates have been found in humans, animals, and the environment, leading to antibiotics' low selectivity. Based on the concept of One Health, we should avoid the outbreak of CP-EC through various methods, including developing bacterial vaccines, originating antibiotic management, and establishing a better medical environment.

## Data availability statement

The datasets presented in this article are not readily available because data is precious scientific research and therefore not publicly available. Requests to access the datasets should be directed to michelleko8688@qq.com.

## Author contributions

YLi and YLv conceived the study. WK, ST, CC, and TL conducted all experiments. WK and CC analyzed the WGS data and drafted subsequent versions. TL, RL, and YZ interpreted the findings. WK and ST wrote the first draft of the manuscript. All authors acquired the data, critically reviewed this report, and approved the final version. All authors have read and agreed to the published version of the manuscript.
