# Corrigendum: Molecular epidemiology and comparative genomics of carbapenemase-producing *Escherichia coli* isolates from 19 tertiary hospitals in China from 2019 to 2020

**DOI:** 10.3389/fmicb.2023.1224271

**Published:** 2023-06-19

**Authors:** Weihsin Ko, Songlu Tseng, Chiahsin Chou, Tianmeng Li, Rose Li, Yaqiao Zhang, Yun Li, Yuan Lv

**Affiliations:** ^1^Institute of Clinical Pharmacology, Peking University First Hospital, Beijing, China; ^2^Department of Trauma and Orthopaedics, Peking University People's Hospital, Beijing, China; ^3^Department of General Surgery, Peking University First Hospital, Beijing, China; ^4^Department of Infectious Diseases and Clinical Microbiology, Beijing Institute of Respiratory Medicine and Beijing Chao-Yang Hospital, Capital Medical University, Beijing, China; ^5^Department of Clinical Medicine, Civil Aviation General Hospital, Beijing, China; ^6^Department of Clinical Medicine, Peking University International Hospital, Beijing, China

**Keywords:** *Escherichia coli*, carbapenemase, molecular epidemiology, genetic characteristics, *bla*_*NDM*_ gene

In the original article, there was a typo error in the Author's List section.

The author's name was incorrectly written as “Wehsin Ko.”

The correct spelling is “Weihsin Ko.”

The authors apologize for this error and state that this does not change the scientific conclusions of the article in any way. The original article has been updated.

